# Building clinical pharmacology laboratory capacity in low- and middle-income countries: Experience from Uganda

**DOI:** 10.4102/ajlm.v12i1.1956

**Published:** 2023-02-07

**Authors:** Denis Omali, Allan Buzibye, Richard Kwizera, Pauline Byakika-Kibwika, Rhoda Namakula, Joshua Matovu, Olive Mbabazi, Emmanuel Mande, Christine Sekaggya-Wiltshire, Damalie Nakanjako, Ursula Gutteck, Keith McAdam, Philippa Easterbrook, Andrew Kambugu, Jan Fehr, Barbara Castelnuovo, Yukari C. Manabe, Mohammed Lamorde, Daniel Mueller, Concepta Merry

**Affiliations:** 1 Infectious Diseases Institute, Makerere University College of Health Sciences, Kampala, Uganda; 2 Department of Medicine, School of Medicine, Makerere University College of Health Sciences, Kampala, Uganda; 3 Department of Clinical Chemistry, University Hospital Zurich, University of Zurich, Zurich, Switzerland; 4 Department of Clinical Research, London School of Hygiene and Tropical Medicine, London, United Kingdom; 5 Department of Human Immunodeficiency Virus, World Health Organization, Geneva, Switzerland; 6 Division of Infectious Diseases, Johns Hopkins School of Medicine, Baltimore, Maryland, United States; 7 Department of Pharmacology and Therapeutics, Trinity College Dublin, Dublin, Ireland

**Keywords:** therapeutic drug monitoring, building laboratory capacity, resource-limited setting, HIV, Uganda

## Abstract

**Background:**

Research and clinical use of clinical pharmacology laboratories are limited in low- and middle-income countries. We describe our experience in building and sustaining laboratory capacity for clinical pharmacology at the Infectious Diseases Institute, Kampala, Uganda.

**Intervention:**

Existing laboratory infrastructure was repurposed, and new equipment was acquired. Laboratory personnel were hired and trained to optimise, validate, and develop in-house methods for testing antiretroviral, anti-tuberculosis and other drugs, including 10 high-performance liquid chromatography methods and four mass spectrometry methods. We reviewed all research collaborations and projects for which samples were assayed in the laboratory from January 2006 to November 2020. We assessed laboratory staff mentorship from collaborative relationships and the contribution of research projects towards human resource development, assay development, and equipment and maintenance costs. We further assessed the quality of testing and use of the laboratory for research and clinical care.

**Lessons learnt:**

Fourteen years post inception, the clinical pharmacology laboratory had contributed significantly to the overall research output at the institute by supporting 26 pharmacokinetic studies. The laboratory has actively participated in an international external quality assurance programme for the last four years. For clinical care, a therapeutic drug monitoring service is accessible to patients living with HIV at the Adult Infectious Diseases clinic in Kampala, Uganda.

**Recommendations:**

Driven primarily by research projects, clinical pharmacology laboratory capacity was successfully established in Uganda, resulting in sustained research output and clinical support. Strategies implemented in building capacity for this laboratory may guide similar processes in other low- and middle-income countries.

## Background

Clinical pharmacology is the study of drugs in humans.^1^ The central dogma of clinical pharmacology is ‘drug concentration determines drug actions’.^2^ Therapeutic drug monitoring (TDM) is the laboratory measurement of the drug concentration in a sample matrix.^3^ One of the aims of measuring drug concentrations in TDM is to adjust drug dose to optimise clinical outcomes and minimise adverse events in hard-to-manage diseases like HIV infection and other infectious diseases.^3,4,5,6,7^ Therapeutic drug monitoring is routinely utilised in developed countries but is used only infrequently in low- and middle-income countries (LMICs).^5,8,9^

Laboratories can measure drug concentrations in different sample matrices using immunoassay platforms^10,11,12^ and chromatographic methods like high-performance liquid chromatography (HPLC) and liquid chromatography-mass spectrometry (LC-MS).^3,9^ Because of its high specificity, HPLC is preferred over immunoassays, and its lower cost and local availability make it the preferred choice over LC-MS in many LMIC laboratories.^13^ However, both HPLC and LC-MS platforms require high technical expertise that may not be available in LMICs, and they have weak supply chains for equipment and spare parts.^12^ Staff training and retention are also more challenging in LMICs due to limited pre-service training opportunities and limited career options. As such, despite evidence of its relevance for specialised patient care in other settings,^5^ TDM is not included in the standard care package within the treatment guidelines outlined by the Uganda Ministry of Health.^14^

The Infectious Diseases Institute (IDI), Makerere University College of Health Sciences, is an HIV centre of excellence located in Mulago National Referral Hospital complex in Kampala, Uganda.^15^ The institute, established through international partnerships, is an academic research centre that commenced operations in 2002. By 2019, through its clinic in Mulago and partners, IDI was supporting 329 335 HIV-positive patients actively receiving antiretrovirals. Laboratory units in IDI included a College of American Pathologists-certified IDI clinical core laboratory and a smaller Translational Research Laboratory that was created in 2007 to develop laboratory research capacity for immunology, molecular biology, microbiology, and clinical pharmacology.

Generally, the need for a functional clinical pharmacology laboratory cannot be overlooked, especially in settings with a high disease burden like LMICs. Efforts to enhance clinical pharmacology laboratory capacity must consider multifaceted needs, including human resources, knowledge building, and infrastructure.^16,17,18^ Across its programming, IDI uses a systematic approach (*Capacity Pyramid*) to highlight gaps in interdependent types of capacity – both personal (e.g. skills) and institutional (e.g. systems) – and inform comprehensive interventions.^19^ Furthermore, collaborative partnerships between institutions in developing and developed countries have been used as a key strategy to address challenges in strengthening laboratory capacity.^9,16,18^ This article describes IDI’s experience with developing capacity by establishing and sustaining a clinical pharmacology laboratory in Uganda.

## Description of the intervention

### Ethical considerations

Ethics committee approval was not required for this research. This research involved no human or animal subjects.

### Repurposing of existing laboratory infrastructure

In a clinical pharmacology laboratory for TDM, the process workflow is critical for assay accuracy and should be considered in the design of the laboratory facility.^20^ Between January 2007 and December 2008, 292.53 square feet of space in the Translational Research Laboratory was repurposed to host the clinical pharmacology laboratory. Two fume extractors (to eliminate toxic chemical fumes for personnel health and safety) and one fume hood (for specific sample processing and storage) were already existent before the laboratory was repurposed. A lighting system akin to natural light was installed to ease visibility.

Strong workbenches made of non-porous material were already available in the acquired Translational Research Laboratory, and these were arranged for smooth workflow and to support the instruments. Air conditioning and air filtration systems were also already installed in the acquired laboratory space; these helped minimise dust exposure and ensure ambient temperature for the instruments and laboratory personnel. Constant laboratory operation was supported by both the national electric power supply and a backup generator installed at the IDI in 2004.

### Laboratory equipment acquisition

**FIGURE 1:**
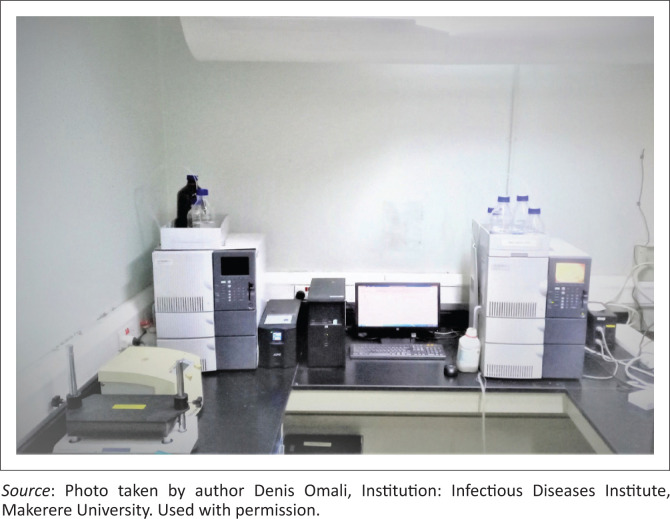
High-performance liquid chromatography-ultraviolet detection machines installed in the clinical pharmacology laboratory at the Infectious Diseases Institute, Makerere University College of Health Sciences, Kampala, Uganda between 2007 and 2013.

Two HPLC-Ultraviolet (HPLC-UV) machines with inbuilt detectors, an autosampler, and pumps (Shimadzu LC-2010CHT, Shimadzu, Kyoto, Japan) controlled by CLASS-VP software version 6.1 (Shimadzu, Kyoto, Japan) were installed in the laboratory (Figure 1). The machines were obtained as generous donations from Trinity College Dublin in 2007 and the University of Zurich in 2013. With internally generated funds, the laboratory acquired other primary instruments like an analytical balance, a pH meter, and a magnetic stirrer. The laboratory also received a generous donation of a centrifuge from the United States National Institutes of Health.

**FIGURE 2:**
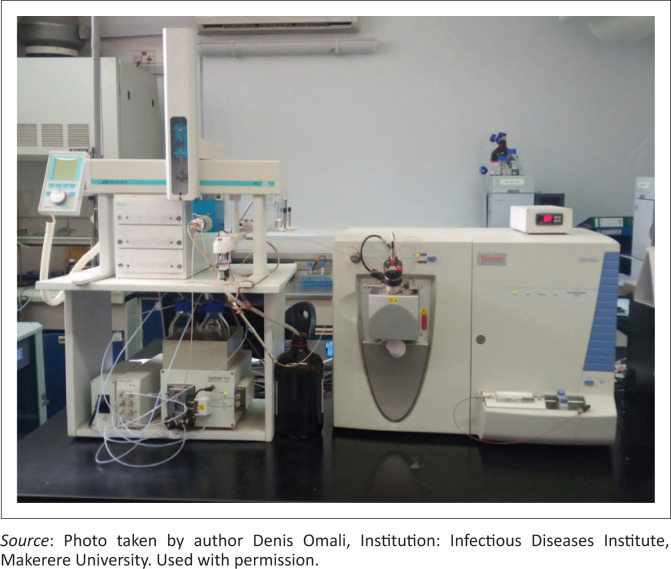
A liquid chromatography-mass spectrometry instrument installed in the clinical pharmacology laboratory at the Infectious Diseases Institute, Makerere University College of Health Sciences, Kampala, Uganda in 2018.

**FIGURE 3:**
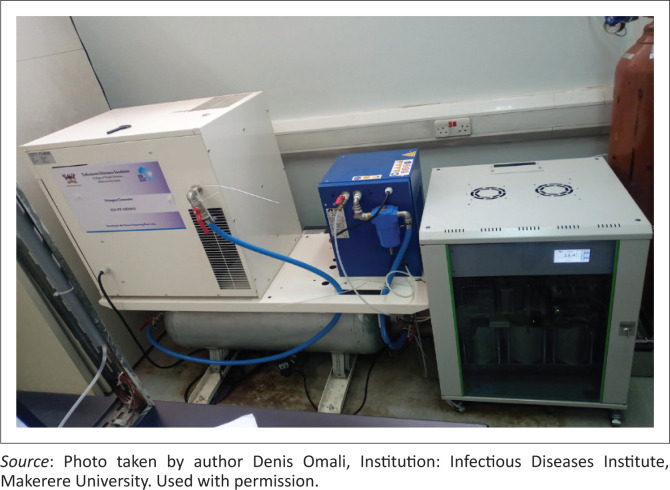
A nitrogen generator installed to support continuous nitrogen gas supply to the mass spectrometer in the clinical pharmacology laboratory at the Infectious Diseases Institute, Makerere University College of Health Sciences, Kampala, Uganda in 2019.

In 2018, leveraging its relationship with the University of Zurich, IDI received a donation of an LC-MS machine (Thermo Scientific LCQ Fleet ion trap LC/MS^n^ model, Thermo Fisher Scientific, San Jose, California, United States), the first of its kind in Uganda for TDM and pharmacological research (Figure 2). Subsequently, in 2019, a nitrogen generator was purchased (Figure 3) to support the operations of the LC-MS. A technical service engineer (originally from South Africa, but subsequently from within Uganda) authorised by the manufacturer services the HPLC-UV instruments biannually. Trained IDI laboratory staff service the LC-MS with expert guidance from the University of Zurich. Also, IDI engineers based at the site oversee the periodic servicing of other laboratory equipment, including the analytical balance, centrifuge, and pH meter.

### Laboratory human resource development

#### Human resource establishment

Quality human resource, which is an important aspect of local capacity, is the centre of most laboratory capacity-building programmes.^16,17,21,22,23^ Establishing quality human resource reduces both appraisal and failure costs, thereby reducing the overall cost of quality, which is a burden in LMICs.^24^ Building on the quality of the Makerere University-Johns Hopkins University collaboration that led to the establishment of a College of American Pathologists-Certified laboratory, two laboratory technologists that had rotated through the IDI clinical core laboratory were recruited.

#### Human resource knowledge building

During the establishment phase, two laboratory technologists were trained in methods for measuring drug concentrations using the HPLC instrument, one at the University of Cape Town and the other at both Radboud University and the University of Zurich. In 2018, through the strong IDI-University of Zurich research collaboration, one staff member was further trained in the use of the LC-MS platform, leading to the donation of the equipment to IDI. The training activities were conducted both physically and via virtual media platforms to expand knowledge and skills in the areas of sample processing and measurement of drug concentrations in different human sample matrices, results analysis, equipment operation and maintenance, method development and validation, and the supply chain process. Using cascade training (Table 1), the formally trained staff member trained five other laboratory technologists to expand the laboratory’s human resource capacity. Continuing medical education was encouraged for the laboratory staff throughout the capacity-building programme at the IDI. Currently, the laboratory has a strong human resource capacity composed of three laboratory technologists and continues to get technical and mentorship support from the University of Zurich.

**TABLE 1:** Human resource training programmes conducted between 2006 and 2019.

Year	Number of staff	Training site	Training period (for external training)	Assay platform
2019††	2	On-site and UZH, Zurich, Switzerland	2 Weeks	LC-MS
2018‡	2§	On-site and UZH, Zurich, Switzerland	1 Mzonth	LC-MS¶
2017‡	1	On-site	-	HPLC-UV
2015‡	2§	On-site and UZH, Zurich, Switzerland	1 Month	HPLC-UV
2014‡	2§	On-site and UZH, Zurich, Switzerland	1 Month	HPLC-UV
2013‡	1	On-site	-	HPLC-UV
2012	1	UZH, Zurich, Switzerland	2 Weeks	HPLC-UV
2011	1	Radboud University, Nijmegen, Netherlands	2 Weeks	HPLC-UV
2011†	1	On-site	1 Month	HPLC-UV
2006	1	University of Cape Town, Cape town, South Africa	1 Month	HPLC-UV

† , Cascade training achieved to the second employee.

‡ , One employee that trained at UZH trained another employee on-site.

§ , One employee trained another employee on-site while training at UZH.

¶ , After attaining required human resource capacity, the LC-MS was acquired through IDI-University of Zurich research collaborative partnership.

†† , Expert visit and training at the clinical pharmacology laboratory at IDI.

UZH, University of Zurich; IDI, Infectious Diseases Institute; LC-MS, liquid chromatography-mass spectrometry; HPLC-UV, high-performance liquid chromatography-ultraviolet detection.

## Lessons learnt

### Current capacity for drug concentration measurements

#### Pharmacokinetic assays

A clinical pharmacology laboratory was developed at IDI and currently has the capacity to measure drug concentrations to guide TDM and clinical pharmacology studies using 10 analytical methods for HPLC-UV and six methods for LC-MS either simultaneously or singly. These methods are used to measure the concentrations of anti-tuberculosis drugs such as ethambutol, rifampicin, isoniazid, pyrazinamide, rifapentine, rifabutin and moxifloxacin. Antiretroviral drugs analysed in the laboratory include nevirapine, efavirenz, atazanavir, lopinavir, tenofovir, saquinavir, darunavir, etravirine, dolutegravir and raltegravir. Antiepileptics (phenytoin and carbamazepine), antibiotics (vancomycin, gentamicin, kanamycin and amikacin), and antimalarial drugs (lumefantrine, artemether and its metabolites and halofantrine) are also analysed in the laboratory.

Currently, the LC-MS methods are used to determine the concentrations of tenofovir, dolutegravir, amikacin, and antimalarial drugs, while the concentrations of other drugs are measured using HPLC-UV. The laboratory staff can also execute in-house innovations to develop, validate, and optimise methods for measuring the concentration of several drugs using the HPLC-UV and LC-MS. Drug concentration data from the laboratory has enabled researchers working on pharmacokinetic studies to detect drug interactions and sub-therapeutic concentrations and monitor patient antiretroviral therapy adherence (Table 2).

**TABLE 2:** Pharmacokinetic studies formally supported by the clinical pharmacology laboratory at the Infectious Diseases Institute in Kampala, Uganda between 2009 and 2015.

Study	Year	Population	Analyte	No. of samples tested
‘Nevirapine pharmacokinetics when initiated at 200 mg or 400 mg daily in HIV-1 and tuberculosis co-infected Ugandan adults on rifampicin.’^25^	2009	HIV-1 and tuberculosis co-infected Ugandan adults on rifampicin	Nevirapine	254
‘Therapeutic drug monitoring of nevirapine in saliva in Uganda using high-performance liquid chromatography and a low cost thin-layer chromatography technique.’^13^	2012	HIV-infected Ugandan adults on nevirapine-based antiretroviral therapy	Nevirapine	587
‘High efavirenz serum concentrations in TB/HIV-co-infected Ugandan adults with a CYP2B6 516TT genotype on anti-TB treatment.’^26^	2013 to 2015	Tuberculosis/HIV-co-infected patients on rifampicin-based anti-tuberculosis therapy and antiretroviral therapy, including 600 mg of efavirenz	Efavirenz	333
‘Study on outcomes related to tuberculosis and HIV drug concentrations in Uganda (South).’^27^	2013 to 2015	HIV/tuberculosis-co-infected adults with a diagnosis of their first episode of pulmonary tuberculosis	Isoniazid, pyrazinamide, rifampicin and ethambutol	6003
‘Antiretroviral concentration measurements as an additional tool to manage virologic failure in resource limited settings: A case control study.’^15^	2015	Patients on any first-line or second-line antiretroviral therapy regimen for at least 6 months	Efavirenz, nevirapine, atazanavir, lopinavir.	573

#### Laboratory quality assurance programme

In 2017, the laboratory commenced its participation in an external quality assurance (EQA) programme for nevirapine, efavirenz, atazanavir, and lopinavir through the *Stichting Kwaliteitsbewaking Klinische Geneesmiddelanalyse en Toxicologie* (KKGT) (Association for Quality Assessment in Therapeutic Drug Monitoring and Clinical Toxicology, Amstelveen, the Netherlands).^28^ The EQA samples analysed in 2017 and 2019 were within the KKGT acceptance range in the four annual rounds. In 2018, EQA test results were within the KKGT acceptance range except for atazanavir in round one, lopinavir in rounds two and three, and efavirenz in round four.

In 2019, anti-tuberculosis drugs, including ethambutol, rifampicin, isoniazid, pyrazinamide, rifapentine, and rifabutin, were included in two annual rounds of the EQA programme. The first attempt yielded results within the KKGT acceptance range in both rounds for all anti-tuberculosis drugs except isoniazid in round two. The second attempt in 2020 was not conducted for anti-tuberculosis drugs due to interruptions in the shipment of the EQA samples to the laboratory.

Antibiotics (only amikacin and vancomycin) and antiepileptics (only phenytoin and carbamazepine) were included in the 2017 EQA subscription, with four annual rounds. Attempts to analyse the antiepileptics in all four rounds yielded results that were outside the KKGT acceptable range. After laboratory preparations, vancomycin was included in the EQA programme in 2020 and yielded results within the KKGT acceptance range in rounds one and four. However, the vancomycin EQA results for rounds two and three were out of the KKGT acceptance range. Amikacin was not tested together with vancomycin in the EQA programme because the method to measure amikacin concentrations in the laboratory was not developed until 2020.

With mutual interdependence with collaborators, the laboratory continues to develop capacity for other KKGT programmes, with continuous optimisation of existing assays for the measurement of drugs like the antiepileptics for which measurement was unsuccessful in the previous attempts. The lack of research studies requiring the measurement of carbamazepine and phenytoin concentrations may have contributed to the lesser focus on the antiepileptics programme. The EQA helped laboratory staff to identify pitfalls in routine laboratory analyses. Interference of other analytes with the KKGT EQA samples was found to be the major cause of out-of-range low scores in the antiretroviral programme. This challenge was corrected by optimising the methods for simultaneous measurement of efavirenz, lopinavir, and atazanavir and using a different analytical method that had no interference between analytes. Generally, participation in the EQA scheme improved staff confidence in supporting research studies and clinicians seeking TDM services.

#### Laboratory support for therapeutic drug monitoring and the need for a clinical pharmacology laboratory

Clinicians and clinical researchers at the IDI clinic and external institutions have successfully used drug concentration results from the laboratory for TDM, specifically to assess non-adherence to regimens or to switch or discontinue patient therapy because of suspected drug resistance and toxicities. Clinicians obtain patients’ blood samples and send the harvested plasma to the clinical pharmacology laboratory for testing with a corresponding request form attached. Results from the laboratory are returned to clinicians to inform clinical management (counselling or dose adjustment, where appropriate). Cumulatively, the laboratory tested 181 clinical care samples from the Adult Infectious Diseases Clinic at the IDI between 2013 and 2019. With this support for clinical management, the need for a clinical pharmacology laboratory cannot be overemphasised, especially in LMICs where there is a high disease burden.

#### Challenges and solutions encountered during laboratory capacity-building processes

The instruments used for drug concentration measurements are costly and were acquired free of charge to IDI through international collaborations (grants and donations). Nevertheless, the costs of equipment preventive maintenance remained a challenge because of the high costs of service vendors and spare parts. The laboratory was able to incorporate these costs within research project budgets over the years, including from competitive grants. Uganda has only a few authorised vendors that supply genuine high-purity reagents of HPLC and LC-MS grade, and these are also costly to procure. Before the acquisition of a nitrogen gas generator, obtaining high-purity gases like nitrogen and helium was difficult. Initial training of laboratory technologists was limited to two staff members since this had to be conducted overseas. Fortunately, efforts to cascade training to other laboratory staff were successful at minimal costs. Notably, these challenges raise the cost of measuring the concentration of a drug in a single sample to approximately $40 United States dollars, which is outside the reach of many patients. From inception, the laboratory’s business case has thus focussed on funded research and clinic projects. For example, the TDM services at the IDI clinic had to be paused in 2019, after analysing 181 samples from clinic patients, due to funding constraints. However, the clinical pharmacology laboratory remained operational since no such laboratory supporting clinical care was established in the country.

## Recommendations

The strategies employed in our laboratory yielded tangible results similar to those observed in related laboratory capacity-building programmes.^16,17,18,22,23,29,30^ Clinical pharmacology laboratory capacity was developed to measure concentrations of antiretrovirals, anti-seizures, antibiotics, antimalarials, and anti-tuberculosis drugs for TDM. The capacity to develop, validate, and optimise analytical methods for other drugs was also developed. The success recorded for this laboratory capacity-building process reflects the gradual progress to strengthen capacity from inception to date with collaborative support. The focus of the institution’s research programme in the field of pharmacokinetics was sustained over the years, enabling continuous cash flow to the laboratory in the form of research costs covering sample analysis. However, with short-term project support as the main source of laboratory resources, factors beyond the laboratory’s direct control such as grant success or availability of collaborators with aligned goals could lead to shocks in the near term and impair long-term sustainability. We therefore recommend that laboratories in LMICs (and their parent institutions) must be prepared to strengthen and sustain results from collaborative programmes when external support ends.

Laboratories in LMICs intending to build and sustain long-term capacity for clinical pharmacology should build local capacity through training and infrastructure development.^16,17,18,31^ As with other capacity-building programmes, staff training in our setting was challenging,^16,31^ requiring international travel and time off work. We further recommend the introduction of practical coursework for clinical pharmacology sessions in pre-service laboratory education (bachelor’s degree-level laboratory technology training) to ease future on-the-job staff training. The African Field Epidemiology Track programme developed and adopted this strategy, positively changing the laboratory profession in its programme member countries.^17,32^ Using established centres like IDI for postgraduate training in pharmacology or for placements to support new laboratories could reduce costs associated with expensive international training. Further, pharmacology postgraduates can be included in laboratory mentorship programmes in LMICs.

Investment in appropriate infrastructure at inception not only promotes staff safety but can also prolong the lifespan of the equipment. High-level air conditioning mitigates challenges of hot and humid climates that may affect laboratory instruments. The use of fume extractors to eliminate environmental dust and toxic fumes from laboratory reagents and processes is essential for staff health and safety. Laboratory fume hoods should be used together with proper reagent segregation to minimise the chances of explosions resulting from flammable reagent fume reactions.

The outcomes presented in this article represent success at a site where clinical research and laboratory infrastructure were already present and operating to international standards at baseline. Our experience may thus not apply to all LMICs due to variations in local guidelines and socio-economic factors. A clinical pharmacology laboratory was established, and laboratory capacity was developed and sustained at the IDI through strong capacity-building collaborative relationships. The laboratory significantly contributed to research capacity development at IDI and other external institutions by providing answers to questions from several pharmacology research studies. Building clinical pharmacology laboratory capacity in LMICs is feasible and necessary to support the global goal of managing HIV infection and other diseases.
